# Characteristics and outcomes of patients admitted to the first psychiatric intensive care unit in Egypt

**DOI:** 10.4102/sajpsychiatry.v27i0.1527

**Published:** 2021-03-18

**Authors:** Tarek A. Okasha, Walaa M. Sabry, Nivert H. Zaki, Menan A. Rabie, Yomna A. Elhawary

**Affiliations:** 1 Department of Neurology and Psychiatry, Faculty of Medicine, Ain Shams University, Cairo, Egypt

**Keywords:** psychiatric units, intensive care, outcomes, demographic-characteristics, psychiatric intensive care units

## Abstract

**Background:**

Psychiatric intensive care units (PICU) have become an essential part of psychiatric hospital design worldwide, there are few published data about their effectiveness.

**Aim:**

In this study, the characteristics and outcomes of 50 Egyptian patients admitted to the first PICU in the Middle East region between April 2015 and October 2018 were retrospectively examined.

**Setting:**

The study was conducted at the Institute of Psychiatry, Ain Shams University.

**Methods:**

Data on patients in PICU at the Institute of Psychiatry, Ain Shams University, were collected retrospectively and analysed and included information on previous psychiatric contact, diagnoses, causes of admission and outcomes. Continuous and categorical variables were subjected to statistical analyses.

**Results:**

The majority of patients in PICU were of female gender, having a diagnosis of schizophrenia. The most common reason for admission to the PICU is the management of delirium, followed by catatonia. The average length of patients’ stays in PICU ranged from half a day to 16 days. Immediate outcome differed where the majority of patients (47 patients, 94%) were discharged to the inpatient psychiatric ward.

**Conclusion:**

This study reviewed practice in the first PICU in Cairo, Egypt, over 3 years, showing the importance of ongoing evaluations of patient populations in providing the best clinical practice; the typical PICU patient is likely to be: female, suffering from schizophrenia or bipolar affective disorder (BAD). The most common reason for PICU admission is for the management of delirium followed by catatonia.

## Introduction

Optimisation of care in an inpatient setting for the mentally ill is one of the challenges facing mental health professionals. Developing psychiatric intensive care units (PICUs) is an approach to enhance the level of care for those patients.^1^

Psychiatric intensive care units were introduced in the 1970s in the United States of America,^2^ the United Kingdom,^3,4^ and in the 1980s in Australia^5,6,7,8^ and Canada.^9^ They were established to provide intensive, short-term, individualised care for those patients who are in an acutely disturbed phase of a serious mental illness.

There are specific criteria for patients’ admission and discharge, and the majority of PICUs have locked doors.^10,11^ Although Rachlin^2^ reported that 20% of his patients stayed over 2 months, nowadays, the average length of stay in different PICUs ranges from 2.6 days^7^ to 30 days.^12^

Various literature report differences between PICUs, with regard to patients’ characteristics, diagnoses, treatment and outcomes. The possible explanation for these differences includes admission policies, traditions in the use of parenteral medications, attitudes towards restraints and seclusions, unit facilities and the number of available beds.

Taking the complexity of the care in PICUs along with the special needs of both the patients and staff into consideration, it is mandatory to conduct research to provide PICU staff from different cultures with a broad and comprehensive knowledge of the ethical, health and social factors affecting their patients and to evaluate the effectiveness of this care.

Up until 2014, there was no detailed history of the use of PICUs in Egypt and because of the need for the so-called ‘special care wards’. The Ain Shams University Institute of Psychiatry (ASUIP) established the first PICU in Egypt and the Middle East. To the knowledge of authors, the current study would be the first one aimed at providing some objective insight related to the characteristics and outcomes of a sample of mentally ill patients who were admitted to the first PICU in Egypt.

## Research methods and design

### Site of the study

This is a retrospective study that was carried out at the Institute of Psychiatry Ain Shams University Hospitals (Egypt). The institute is located in Eastern Cairo, and its outpatient clinics provide psychiatric services to a catchment area of about the third of greater Cairo from both urban and rural areas. The hospital runs a service of five general psychiatry clinics per day for 4 days and three clinics of drug dependence management per day for 2 days.

When the service commenced at ASUIP in 1990, there were 81 inpatient adult ward beds. Nowadays, the inpatient service consists of a 120 adult-bed unit and a two-bed PICU without seclusion rooms.

### Participants and setting

The sample included all of the different consecutive patients admitted from 01 April 2015 to 31 October 2018 **(***n* = 50**)** to the PICU of ASUIP. The only exclusion criterion was a hospital stay of less than 24 h as the patient is not adequately assessed and investigated.

## Ain Shams University Institute of Psychiatry’s psychiatric intensive care units

Psychiatric intensive care units at ASUIP are a two-bed unit, run by specialised nursing staff in emergency psychiatry. Staff to patient ratios were a minimum of 1:1, unlike other ward areas where staff to patient ratios are much lower, and the PICU did not have a seclusion room. The unit was established to deal with patients with challenging behaviours and those with serious mental illness who could not be managed safely on less staffed wards. Decisions for transfer to and from the PICU were made by specialist psychiatrists from the relevant areas. The unit has specific admission and discharge criteria in terms of patients’ clinical characteristics.

The philosophy of the PICU is to provide a modern, needs-led mental health service, which treats patients with dignity and respect in an appropriate environment that is safe and supportive for patients and staff. The belief that individuals with mental illness are violent, unpredictable and dangerous is a pervasive stigmatising view, which has been shown to negatively affect perceptions of personal safety in general medical wards in our university hospital, in addition to the fact that patient developing condition such as national minimum standards (NMS) or delirium needs quick intervention rather than long waiting time for a vacant bed in ICU dealing with general medical conditions; this makes the availability of a place quite a challenge.

In line with the NMS for PICU and low secure environments^13^ the admission criteria include the following: adult psychiatric inpatients who developed a new symptom that cannot be controlled in the ward and needs emergency short-term intervention including *extrapyramidal symptoms* (as detected by Simpson Angus Scale > 14),^14^ *akathisia* (by Barnes Akathisia Rating Scale [BARS] Global > 2, objective + subjective > 6),^15^ *acute dyskinesia* (by Abnormal Involuntary Movement Scale [AIMS] > 12),^16^ *acute dystonia* (confirmed by clinical examination), *agitation/sedation* (by Richmond Agitation Sedation Scale [RASS] Between –3 and +3),^17^ *catatonia* (by Bush–Francis Catatonia Rating Scale [BFCS]),^18^ *neuroleptic malignant syndrome* (Mini Mental State Examination [MMSE] < 22),^19^ *delirium* (by Delirium Observation Screening Scale [DOS] > 6),^20^ confusion (MMSE < 22) and serious suicidal attempt (by Pierce Suicide Intent Scale).^21^

Psychiatric intensive care units’ team members planned to conduct an intervention to improve patient privacy and satisfaction, including PICU environmental space organisation (two isolated beds), process management, access control, staff education and training and encouragement of ethics consultation. The intervention was discussed and initiated with the support of senior hospital administrators and the Ain Shams University’s quality assurance committee.

The PICU team has also developed various management algorithms and/or protocols (four training courses were held for residents and young doctors as well as two training sessions to qualified nurses who had previous experience in dealing with emergency situations, two audits also took place as part of *auditing the services provided in different units of institute of psychiatry*, documentation reviewing weekly and monthly, 3 months audit for evaluation of the process, in addition to a ‘Drinking water campaign’ encouraging good hydration to all patients to minimise the rising number of delirium cases in hot climates), which provided a framework for the unit staff to manage patients in a reasoned, practical, efficacious and person-centred manner. Changes and adaptation to algorithms and/or protocols were achieved gradually over 3 years from 2015 to 2018.

### Data collection

Data were extracted from ASUIP clinical data sheets of patients who were admitted to ASUIP PICU between its opening in April 2015 and October 2018.

The collected data on the demographic, medical and/or psychiatric history of all subjects and details pertaining to their current admission, for example presenting problems, management problems and outcome following the PICU admission, were examined. Psychiatric diagnoses of these patients were based on DSM-V criteria.^22^

### Ethical considerations

The study was conducted in accordance with the Helsinki Declaration for medical research of 1975 and in compliance with the guidelines of the Research and Ethics committee of Institute of Psychiatry, Ain Shams University. Patients were informed and signed an informed consent that their data would be used in future research in complete confidentiality from their medical records.

## Result

During the study period, there were 50 patients admitted to PICU. Twenty-four patients were male (48%) and 26 were female (52%). Their mean age was 37.9 years (± 15.7) with an age range of 14–77 years.

## Clinical characteristics of the study sample

### Psychiatric and/or medical diagnosis

According to DSM-V diagnostic criteria,^22^ the majority of patients admitted to PICU (22 patients, 44%) were diagnosed with schizophrenia, whilst 13 patients (26%) were having bipolar disorder. The third diagnosis was acute polymorphic psychosis, comprising 6 patients (12%). Four of patients (8%) were diagnosed with severe major depressive disorder.

With regard to medical co-morbidities, ten (20%) of patients had medical co-morbidities in the form of cancer thyroid and lung metastases, diabetes mellitus, anaemia, hepatosplenomegaly, hypertension, systemic lupus erythematosus (SLE), hip fracture, cerebrovascular stroke, deep venous thrombosis (DVT), epilepsy and paraparesis. Detailed data are illustrated in Table 1.

**TABLE 1:** Characteristics of the sample.

Variables	Number	%
**Demographic**		
Male	24	48
Female	26	52
**Diagnosis**		
Schizophrenia	22	44
Bipolar affective disorder	13	26
Acute polymorphic psychosis	6	12
Major depressive disorder	4	8
Schizoaffective disorder	2	4
Anorexia nervosa	1	2
Dementia	1	2
Substance-induced mood	1	2
**Co-morbidities**		
Psychiatric co-morbidity substance abuse	1	2
Lithium toxicity	1	2
Intellectual disability IQ (50)	2	4
Medical co-morbidity cancer thyroid with lung metastasis	1	2
Diabetes mellitus	2	4
Cerebrovascular stroke	2	4
Deep venous thrombosis	1	2
Anaemia	2	4
Hepatosplenomegaly	1	2
Systemic lupus erythematosus (SLE)	1	2
Hip replacement	1	2
Paraparesis	1	2
**Comminuted fracture in L2 vertebral body 1 (2)**		
Left parietal hematoma	1	2
Epilepsy	1	2

### Causes of patients’ transfer to psychiatric intensive care units

Patients’ referral to the PICU usually followed a deterioration in their psychiatric and/or medical conditions. Nineteen patients’ (38%) admissions were owing to developing delirium because of psychiatric causes (like lithium toxicity, delirium on top of dementia), 16 patients (32%) were because of developing catatonic features, three patients (6%) were transferred to PICU because of respiratory problems and two patients (4%) because of developing electrolyte disturbances. Other causes are illustrated in Table 2.

**TABLE 2:** Causes of admission.

Variables	Number	%
Delirium because of psychiatric cause	19	38
Catatonia	16	32
Neuroleptic malignant syndrome	1	2
One-to-one observation for suicidality	1	2
Blood transfusion for anaemia	1	2
Receiving pulse steroids in systemic lupus erythematosus (SLE)	1	2
Post-operative monitoring	2	4
Vital data monitoring	-	-
Uncontrolled atrial fibrillation (AF), haemodynamic instability, blood pressure control	3	6
Receiving proton pump inhibitor (pantoprazole) infusion	1	2
Respiratory problems	3	6
Electrolyte disturbance	2	4

Our results showed that 74% of patients transferred to PICU were because of worsening of their mental health problems (delirium, neuroleptic malignant syndrome, suicidality, catatonia), whilst the other 26% of patients were admitted because of deteriorating medical problems (Table 2).

### Length of stay at psychiatric intensive care units

Mean duration of PICU care was 5 (± 3.45) days with the least duration half a day and the maximum duration 16 days.

### The incidence and management of dangerous and disruptive behaviour during PICU admission

Although aggression was not amongst the main reasons for admission to our PICU, one patient (2%) showed agitation frequently during his PICU stay as confirmed by RASS. The containment methods used for this patient during his PICU care were as follows: medications (coerced, rapid tranquillisation or PRN and special observation).

### Outcome after discharge from psychiatric intensive care units

After receiving appropriate management for every case, immediate outcome differed where 94% of patients (47 patients) were discharged to the inpatient psychiatric ward, 2% of patients (1 patient) were transferred to the stroke unit because of suspected cerebrovascular incident, 2% of patients (1 patient) were transferred to ICU, the patient with neuroleptic malignant syndrome was discharged to the inpatient psychiatric ward with improvement in all clinical and rating scales data. One patient (2%) was about to be transferred to ICU; however, she had a cardiac arrest and died.

## Discussion

Data from previous studies confirmed the utmost need for healthcare professionals to provide objective measurements of patient care.^23^

A paucity of research documenting characteristics of PICU populations exists, possibly reflecting heterogeneous admission criteria and the relatively recent introduction of such units.

In this study, 50 patients were included from ASUIP clinical datasheets between 2015 and 2018.

Findings of previous studies emphasised that the majority of patients admitted to the PICU were assessed as being at a high level of risk either to themselves or others.^23^ This finding is in line with our unit’s policies and admission criteria, where the assessment of patients’ eligibility to be transferred to ASUIP’s PICU was carried out both subjectively and objectively by using specific psychometric and clinical scales.

The majority of studies give a mean age for patients at the PICU in their 30s,^2,24^ which is slightly lower than the mean ages of our patients (37.9 ± 15.7). Whilst other studies showed similar patients’ age to ours.^25^ The lower ages of participants in the given studies compared with our study could be because of different admission criteria as the most common reason for admission to their PICU is aggression management, followed in rank order by generally disruptive behaviour, which is more prevalent in younger ages.^26,27,28^

Specific gender representation in the PICU admissions varies between different studies. In our study, the majority of admitted patients were female (52%). This is inconsistent with most of the previous studies which demonstrated that the majority of their PICU patients are males.^29,30,31^ One possible explanation for this sex difference in PICU risk proved to be because of unmeasured confounders in some studies (like limited access for females to PICU beds),^32^ and some case–control studies have typically failed to observe such sex differences.^33^

Our report showed that most patients admitted to ASUIP’s PICU were diagnosed with schizophrenia (44%) followed by bipolar disorder (32%). These findings are consistent with those identified by different studies, which suggested that a high proportion of admitted patients to PICUs were disturbed patients with psychotic and affective disorders.^29,31,34^ This could be attributed to the nature of the majority of cases admitted to Egyptian mental hospitals, as a recent study by Okasha found that 79% of patients in a mental hospital were having a diagnosis of the schizophrenic spectrum.^35^ This finding highlights the importance of PICU staff making accurate predictions of and having organised management plans to deal with such patients.

However, this finding is in contrast to other studies, which demonstrated that patient diagnosis was unrelated to PICU transfer with the exception that patients with bipolar disorder were twice as likely to be transferred to PICU compared with patients with schizophrenia.^36,37^ This could be because of different PICU policies and admission criteria. As in the above studies, their main admission criteria are to deal with aggression, which is greater in bipolar disorder than in schizophrenia and other psychiatric disorders.^38,39^

We found that the majority of admissions to the PICU (74%) were as a result of a significant deterioration in mental state or behaviour of patients. Patients with delirium represented the highest percentage (38%) followed by those presented with catatonic features (32%). Our report is different from other studies that revealed that aggression is the most common cause for transferring patients to PICU, which accounts for 30% – 50% of admissions, and disruptive or acutely psychotic behaviours are the next most common reason for admission, accounting for 10% – 20% of admissions.^40^ Other studies showed suicide risk^41^ and risk of absconding as the primary reason.^2^ Differences could be because of differences in the admission criteria and objective measurements used in different PICUs.

High delirium rates amongst admitted PICU patients could be explained by the high trend of using polypharmacy in Egyptian patients,^42^ and the climatic changes in Egypt, which render patients more susceptible to dehydration and electrolyte disturbance.

Beside clinical assessment by a senior psychiatrist, diagnostic confirmation for catatonia was performed by using a sensitive, standardised rating instrument for catatonia (BFCS); catatonia is a common presentation amongst mentally ill patients from developing countries in comparison with the western population especially for schizophrenic spectrum disorders.^43,44,45^

In our results, there were no cases admitted to PICU because of agitation or violence. Over the past few years, there have been innovations and changes to PICU provisions from just a seclusion unit to a larger-scale service unit that provides management of acute mentally ill patients.^46^ This finding is in line with our admission criteria developed by PICU staff. It also denotes that the staff in the acute inpatient ward had good expertise and confidence to communicate with and manage potentially aggressive and highly aroused patients. Significant differences and similarities in the operation of the psychiatric units were found. Divergences can be explained and justified by differences in patient characteristics and differences in the organisational culture of the mental healthcare institutions.

However, our finding revealed that one-quarter of patients were admitted to the PICU owing to physical healthcare needs, because it provided a safe environment, that is high levels of staffing and observation. This phenomenon probably reflects the high needs of PICU staff for proper training on management of common medical health problems and to liaise closely with other practitioners.

The average length of stay in our PICU ranged from half a day to 16 days during the study period. Most studies reported a wide range of lengths of stay. This ranged from a very high length of stay up to 51 days,^9^ 131 days,^29^ 365 days,^47^ ‘many months’,^12^ ‘more than a month’,^48^ whereas other studies showed a lower range of PICU stay of less than 7 days.^1,49^ This variability in results could be explained by the fact that longer staying patients could be possibly associated to some degree with forensic issues.

With regard to the immediate outcome of patients after receiving their appropriate management, 94% of patients^47^ were discharged to other wards, 2% of patients^1^ were transferred to stroke unit because of the suspected cerebrovascular incident and 2% of patients^1^ were transferred to ICU. In comparison with a study by Dolan and Lawson^50^ who demonstrated that amongst 73 PICU admissions, 51 patients (65%) had moved to other clinical areas within the unit and surprisingly 4 cases (5%) were discharged to their family home, whilst 2 (3%) to hostel accommodation and 5 (6%) to district psychiatric hospitals, 9 (12%) cases required to transfer to maximum security owing to escalating violence in the PICU and 3 (4%) sentenced prisoners were returned to prisons following stabilisation on medication.^50^ Factors that determine the difference between studies are the difference in discharge criteria where, in ASUIP PICU, patients are transferred upon objective evaluation and clinician satisfaction of the patients’ suitability to be discharged from PICU.

## Conclusion

This study reviewed practice in the first established PICU in Cairo, Egypt, over 3 years. This study shows the importance of ongoing evaluations of patient populations in providing the best clinical practice in psychiatric care. In comparison with acute mentally ill patients, the typical PICU patient is more likely to be female and suffering from schizophrenia or bipolar affective disorder (BAD). The most common reason for admission to the PICU is the management of delirium, followed by catatonia or any other emergency condition that may occur to psychiatric patients and requires intervention in a specialised unit. From there came the idea of slightly changing the wording to psychiatric intermediate care unit as it encompasses more cases than the ordinary PICU.

In addition, PICUs are a major part of psychiatric care provision but have not received enough substantive, grant-funded research attention. So further studies on different management protocols that are followed in such units and evaluation of their cost-efficacy, comparing them with acute wards, are needed. Patients’ and staff’s satisfaction of PICU policies are to be addressed, in addition to enhancing the continuity of documentation of patients’ clinical data, medications or procedures provided and outcomes.

Care plans should incorporate aspects of specific cultural, ethnic, gender-specific care provision to facilitate needs where possible. The care plans should be recovery-focussed and sensitive to aspects and protected characteristics of the individual, and this were applied in our unit as having a two isolated bed unit preserving cultural and religious issues of having a male and female patient admitted at the same time ensuring the privacy of each. Sometimes visits of religious men are arranged, which improves patients’ recovery and well-being.

### Clinical implications

This study shows the importance of continuous assessment of patient populations in promoting best practice guidelines in psychiatric care. It reveals the need to conduct future studies on different management protocols used in the PICU and training of psychiatrists on emergency medical conditions to deal with critical psychiatric patients in the PICU.
